# Supplementary material for: An Initial Survey on Occurrence, Fate, and Environmental Risk Assessment of Organophosphate Flame Retardants in Romanian Waterways
Source: J Xenobiot. 2023 Dec 22;14(1):31–50. doi: 10.3390/jox14010003 (PMC10801549; doi:10.3390/jox14010003)
Supplement: Supplementary file 1 [file jox-14-00003-s001.zip › jox-2726107-supplementary.pdf]

Supplementary Materials: An Initial Survey on Occurrence, Fate, and Environmental Risk Assessment of Organophosphate Flame Retardants in Romanian Waterways

Iuliana Paun, Florinela Pirvu, Vasile Ion Iancu, Marcela Niculescu, Luoana Florentina Pascu and Florentina Laura Chiriac

National Research and Development Institute for Industrial Ecology—ECOIND, Drumul Podu Dambovitei Street 57-73, 060652 Bucharest, Romania

Tabel S1. Collection points and sample codification

City	WWTPs					Natural receivers		
	WWTPs codification	Population	Daily flow (m^3/zi)	Sample type	Sample code	River	Sample type	Sample code
Targoviste	S1	79600	47606	Influent	IF1	Ialomita	Upstream	AM1
				Effluent	EF1		Downstream	AV1
				Sludge	N1			
Galati	S2	504000	224640	Influent	IF2	Siret	Upstream	AM2
				Effluent	EF2		Downstream	AV2
				Sludge	N2			
Glina	S3	1830000	1028160	Influent	IF3	Dambovita	Upstream	AM3
				Effluent	EF3		Downstream	AV3
				Sludge	N3			
Iasi	S4	793500	777600	Influent	IF4	Bahlui	Upstream	AM3
				Effluent	EF4		Downstream	AV3
				Sludge	N4			
Ramnicu Valcea	S5	110527	88128	Influent	IF5	Olt	Upstream	AM4
				Effluent	EF5		Downstream	AV4
				Sludge	N5			

Table S2. Physical-chemical properties of the OPFR compounds

Nr. Crt.	Name	Abrev	CAS No.	Formula	MW[a]	Log K_{ow}	Log K_{oc}	Water solubility (mg/L)	BCF
1	Tris (2-chloroethyl) phosphate	TCEP	115-96-8	$C_6H_{12}Cl_3O_4P$	285.49	1.63	2.48	878	0.42
2	Tripropyl phosphate	TPP	513-08-6	$C_9H_{21}O_4P$	224.23	1.87	2.83	827	0.91
3	Dibutyl phosphate	DBP	838-85-7	$C_8H_{19}O_4P$	210.21	2.29	2.18	3830	1.92
4	Tris (1-chloro-2-propyl) phosphate	TCPP	13674-84-5	$C_9H_{18}Cl_3O_4P$	327.57	2.59	2.21	1600	7.94
5	Tris(1,3-dichloro-2-propyl) phosphate	TDCPP	13674-87-8	$C_9H_{15}Cl_6O_4P$	430.90	3.65	3.96	1.50	-
6	Tris (2,3-dibromopropyl) phosphate	TDBPP	126-72-7	$C_9H_{15}Br_6O_4P$	697.61	3.71	3.40	8.0	21.4
7	Tris (2-ethylhexyl) phosphate	TEHP	78-42-2	$C_{24}H_{51}O_4P$	434.63	9.49	6.36	0.0003	3.16
8	Triphenyl phosphate	TPHP	115-86-6	$C_{18}H_{15}O_4P$	326.28	4.59	3.72	1.03	113.3
9	Tricresyl phosphate	TMPP	1330-78-5	$C_{21}H_{21}O_4P$	368.36	6.34	4.34	0.018	2534
10	Bis(2-ethylhexyl) phosphate	BEHP	298-07-7	$C_{16}H_{35}O_4P$	322.42	6.07	4.23	0.06	49.5
11	Diphenyl phosphate	DPHP	838-85-7	$C_{12}H_{11}O_4P$	250.18	2.88	2.08	82.4	5.44

Table S3. Condition of instrumental analysis for OPFR compounds

Chromatographic column	Zorbax Eclipse Plus C18 (150 x 2.1 mm, 3.5 μm)
Column temperature	40°C
Injection volume	10 μl
Mobile phase	0.1% formic acid in water (A) / 0.1% formic acid in methanol (B)
Mobile phase flow rate	0.2 mL/min
Sample solvent	Water
Elution type	gradient
Chromatographic run-time	35 min

Table S4. Gradient elution program

Time (min)	Mobile phase B (%)
0 - 0.50	60
0.51 - 15.00	90
15.01 - 20.00	100
20.01 - 25.00	100
25.01	60
35.00	Stop time

Table S5. Acquisition time-segments

Time segment	Start Time (min)	Scan type	Div Valve	Store
1	0	MRM	To Waste	No
2	6	MRM	To MS	Yes
3	10	MRM	To MS	Yes
4	13	MRM	To MS	Yes
5	15.8	MRM	To MS	Yes
6	18.8	MRM	To MS	Yes
7	20.3	MRM	To MS	Yes
8	22.4	MRM	To MS	Yes
9	25	MRM	To Waste	No
10	26	MRM	To MS	Yes
11	29	MRM	To Waste	No

Table S6. MRM conditions of OPFR (Q – quantifier, q - qualifier)

Compounds	tR (min)	MRM P → Q / P → q	Fragmentor (V)	CE (V)	CAV (V)	Dwell time (msec)	ESI
DPHP	7.05	249.0→155.0	145	20	0	125	Negative
		249.0→93.2	145	30	1	125	Negative
DBP	7.56	209.2→153.0	120	10	4	125	Negative
		209.0→79.0	120	25	6	125	Negative
TCEP	11.48	285.0→99.0	110	25	5	250	Positive
		285.0→63.0	110	36	5	250	Positive
TPP	14.01	225.1→98.9	95	15	3	250	Positive
		225.0→141.0	95	5	4	250	Positive
TCPP	16.18	327.0→175.0	90	10	2	250	Positive
		327.0→99.0	100	25	4	250	Positive
TDCPP	19.80	432.9→99.1	125	30	1	250	Positive
		430.9→99.1	125	30	1	250	Positive
TPHP	20.57	327.1→215.0	165	25	4	175	Positive
		327.1→152.1	165	60	1	175	Positive
BEHP	20.93	321.3→79.2	190	35	2	175	Negative
TDBPP	22.93	698.6→98.9	135	25	5	125	Positive
		696.6→98.6	150	25	5	125	Positive
TMPP	23.52	369.1→91.0	185	45	3	125	Positive
		369.0→243	185	30	3	125	Positive
TEHP	26.75	435.4→98.9	125	15	4	250	Positive
		435.4→71.0	125	20	3	250	Positive

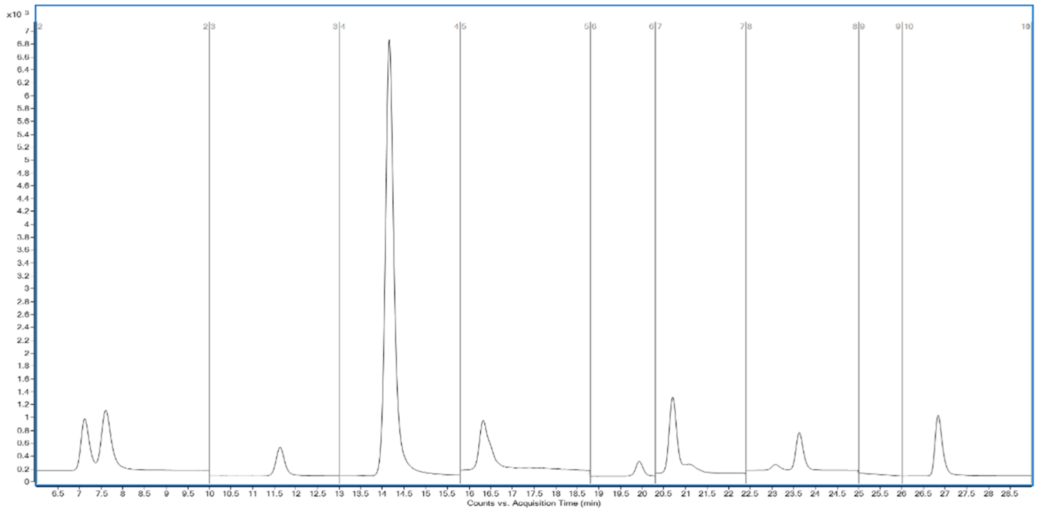

Figure S1. MRM chromatogram registered for a standard mixture of 1 μg/L

Tabel S7. RSD values for intra-day and inter-day precision obtained for a sike concentration of 1 μg/L in each environmental matrix.

Analytes	RSD%								
	Instrument repeatability			Intra-day precision			Inter-day precision		
	Surface water	Wastewater	Sludge	Surface water	Wastewater	Sludge	Surface water	Wastewater	Sludge
DPHP	0.78	0.83	1.19	3.94	4.23	5.35	8.74	9.37	11.2
DBP	0.44	0.65	1.26	4.59	5.16	6.66	8.53	10.1	11.4
TCEP	0.93	1.04	1.12	4.39	5.28	7.15	9.45	11.3	12.6
TPP	0.85	1.98	1.35	5.04	5.79	7.31	9.94	10.8	11.2
TCPP	0.81	0.96	1.02	4.36	5.44	6.72	7.22	8.64	10.0
TDCPP	0.90	1.04	1.27	4.23	4.96	5.68	8.39	9.28	10.3
TPHP	0.79	0.83	1.05	6.65	6.83	7.53	9.26	9.79	11.2
BEHP	0.57	0.72	0.83	3.83	4.25	6.44	7.14	8.85	10.2
TDBPP	0.66	0.81	0.95	4.17	5.17	6.81	8.85	9.66	11.0
TMPP	0.82	0.95	1.04	5.20	6.22	7.12	9.06	10.3	11.7
TEHP	0.74	0.87	1.08	3.96	4.31	5.23	6.16	8.25	10.1

Table S8. Recovery values obtained for all types of matrices (spike concentration = 1µg/L, n = 3)

Analytes	Recovery (%)									
	Upstream	±SD	Downstream	±SD	Effluent	±SD	Influent	±SD	Sludge	±SD
DPHP	84	2.69	81	2.59	79	3.40	76	3.27	79	3.87
DBP	92	2.94	89	2.85	83	3.57	79	3.40	76	3.72
TCEP	88	2.82	87	2.78	81	3.48	73	3.14	75	3.68
TPP	86	2.75	82	2.62	83	3.57	81	3.48	84	4.12
TCPP	89	2.85	84	2.69	80	3.44	88	3.78	88	4.31
TDCPP	105	3.36	109	3.49	76	3.27	83	3.57	75	3.68
TPHP	91	2.91	83	2.66	78	3.35	89	3.83	78	3.82
BEHP	89	2.85	86	2.75	78	3.35	76	3.27	84	4.12
TDBPP	85	2.72	86	2.75	90	3.87	83	3.57	86	4.21
TMPP	92	2.94	82	2.62	91	3.91	87	3.74	114	5.59
TEHP	88	2.82	77	2.46	88	3.78	84	3.61	78	3.82

Table S9. Matrix effects (ME) values determined for the targeted analytes in all environmental samples (post-extraction spike = 1 µg/L, n = 3)

Analytes	EM, %									
	Upstream	±SD	Downstream	±SD	Effluent	±SD	Influent	±SD	Sludge	±SD
DPHP	89	3.03	74	2.52	81	3.73	99	4.55	84	4.12
DBP	98	3.33	81	2.75	70	3.22	80	3.68	73	3.58
BEHP	58	1.97	75	2.53	74	2.52	77	3.54	74	3.63
TCEP	90	3.06	80	2.72	77	3.54	100	4.60	80	3.92
TPP	94	3.20	74	2.52	84	3.86	101	4.65	91	4.46
TCPP	110	3.74	115	3.91	82	3.77	94	4.32	74	3.63
TDCPP	96	3.26	85	2.89	86	3.96	93	4.28	85	4.17
TPHP	79	2.69	79	2.69	86	3.96	75	3.45	90	4.41
TDBPP	88	2.99	89	3.03	98	4.51	56	2.58	88	4.31
TMPP	98	3.33	99	3.37	97	4.46	71	3.27	121	5.93
TEHP	96	3.26	72	2.45	90	4.14	92	4.23	84	4.12

Table S10**.** Instrumental limit of quantitation (IOQ), and method limit of detection and quantitation obtained for the targeted compounds

Analytes	IOQ	Upstream		Downstream		Effluent		Influent		Sludge	
		LOD	LOQ	LOD	LOQ	LOD	LOQ	LOD	LOQ	LOD	LOQ
	µg/L	ng/L								ng/g	
DPHP	0.11	0.21	0.59	0.23	0.64	0.24	0.67	0.28	0.78	0.10	0.28
DBP	0.11	0.19	0.53	0.24	0.67	0.29	0.81	0.31	0.87	0.11	0.32
TCEP	0.09	0.34	0.95	0.31	0.87	0.3	0.84	0.33	0.92	0.09	0.26
TPP	0.06	0.21	0.59	0.13	0.36	0.15	0.42	0.18	0.50	0.06	0.16
TCPP	0.09	0.2	0.56	0.22	0.62	0.2	0.56	0.24	0.67	0.07	0.20
TDCPP	0.15	0.17	0.48	0.24	0.67	0.34	0.95	0.37	1.04	0.15	0.42
TPHP	0.10	0.2	0.56	0.21	0.59	0.22	0.62	0.25	0.70	0.09	0.26
BEHP	0.28	0.24	0.67	0.27	0.76	0.27	0.76	0.31	0.87	0.23	0.67
TDBPP	0.22	0.21	0.59	0.23	0.64	0.25	0.70	0.3	0.84	0.18	0.51
TMPP	0.12	0.19	0.53	0.22	0.62	0.26	0.73	0.28	0.78	0.07	0.21
TEHP	0.07	0.2	0.56	0.17	0.48	0.19	0.53	0.19	0.53	0.07	0.19

Table S11. Acute toxicities (LC50 and EC50) used to calculate risk quotients

OPFRs	Aquatic organisms	Biota	End point	Conc. effect (mg/L)	References
TCEP	Fish	Zebrafish	LC_{50}	202	[1]
	Fish	Carassius auratus	$L(E)C_{50}$	90	[2]
	Daphnia	Daphnia magna	EC_{50}	381	[3]
	Algae	Scenedesmus subspicatus	EC_{10}	6500	[4]
TDCPP	Fish	Zebrafish	LC_{50}	0.42	[1]
	Fish	Carassius auratus	$L(E)C_{50}$	5.1	[2]
	Fish	Oncorhynchus mykiss	$L(E)C_{50}$	1.2	[2]
	Daphnia	Daphnia magna	EC_{50}	7.76	[3]
	Algae	Pseudokirchnerialla subcapitata	$L(E)C_{50}$	39	[2]
TCPP	Fish	Zebrafish	LC_{50}	13.5	[1]
	Fish	Poecilia reticulata	$L(E)C_{50}$	30	[2]
	Daphnia	Daphnia magna	EC_{50}	81	[3]
	Algae	Scenedesmus subspicatus	$L(E)C_{50}$	45	[2]
TPHP	Fish	Zebrafish	LC_{50}	1.03	[1]
	Fish	Oncorhynchus mykiss	$L(E)C_{50}$	0.42	[2]
	Daphnia	Daphnia magna	EC_{50}	1.7	[3]
	Algae	Ankistrodesmus falcatus	EC_{10}	16	[2]
	Algae	Scenedesmus quadricauda	$L(E)C_{50}$	0.5	[2]
TEHP	Daphnia	Daphnia magna	EC_{50}	0.74	[3]
TMPP	Daphnia	Daphnia magna	EC_{50}	0.31	[3]
	Algae	Gasterosteus aculeatus	NOEC	3.2	[2]
TPrP	Fish	Zebrafish	LC_{50}	252	[1]
TDBPP	Fish	Salmo gairdneri	$L(E)C_{50}$	0.516	[5]
	Daphnia	Daphnia magna	$L(E)C_{50}$	4.568	[5]
	Algae	Scenedesmus abundans	$L(E)C_{50}$	0.545	[5]

Table S12.Environmental risk of OPFRs for aquatic organisms

OPFRs	Aquatic organisms	Biota	AF	PNEC (μg/L)	RQ AM	Risk	RQ AV	Risk
TCEP	Fish	Zebrafish	1000	202	1.09×10^{-4}	Low	6.98×10^{-4}	Low
	Fish	Carassius auratus	1000	90	2.44×10^{-4}	Low	1.57E-03	Low
	Daphnia	Daphnia magna	1000	381	5.77×10^{-5}	Low	3.70×10^{-4}	Low
	Algae	Scenedesmus subspicatus	10	65	3.38×10^{-4}	Low	2.17×10^{-3}	Low
TDCPP	Fish	Zebrafish	1000	0.42	2.62×10^{-2}	Low	0.45	Moderate
	Fish	Carassius auratus	1000	5.1	2.16×10^{-4}	Low	1.20×10^{-2}	Low
	Fish	Oncorhynchus mykiss	1000	1.2	9.17×10^{-3}	Low	5.08×10^{-2}	Low
	Daphnia	Daphnia magna	1000	7.76	1.42×10^{-3}	Low	7.86×10^{-3}	Low
	Algae	Pseudokirchnerialla subcapitata	1000	39	2.82×10^{-4}	Low	1.56×10^{-3}	Low
TCPP	Fish	Zebrafish	1000	13.5	6.91×10^{-2}	Low	0.12	Moderate
	Fish	Poecilia reticulata	1000	30	3.11×10^{-2}	Low	5.34×10^{-2}	Low
	Daphnia	Daphnia magna	1000	81	1.15×10^{-2}	Low	1.98×10^{-2}	Low
	Algae	Scenedesmus subspicatus	1000	45	2.07×10^{-2}	Low	3.56×10^{-2}	Low
TPHP	Fish	Zebrafish	1000	1.03	7.18×10^{-3}	Low	9.13×10^{-2}	Low
	Fish	Oncorhynchus mykiss	1000	0.42	1.76×10^{-2}	Low	2.24×10^{-2}	Low
	Daphnia	Daphnia magna	1000	1.7	4.35×10^{-3}	Low	5.53×10^{-3}	Low
	Algae	Ankistrodesmus falcatus	10	0.16	4.63×10^{-2}	Low	5.88×10^{-2}	Low
	Algae	Scenedesmus quadricauda	1000	0.5	1.48×10^{-2}	Low	1.88×10^{-2}	Low
TEHP	Daphnia	Daphnia magna	1000	0.74	4.19×10^{-2}	Low	1.81×10^{-2}	Low
TMPP	Daphnia	Daphnia magna	1000	0.31	6.13×10^{-2}	Low	5.19×10^{-2}	Low
	Algae	Gasterosteus aculeatus	10	0.032	0.59	Moderate	0.50	Moderate
TPP	Fish	Zebrafish	1000	252	4.76×10^{-6}	Low	3.97×10^{-6}	Low
TDBPP	Fish	Salmo gairdneri	1000	0.516	8.72×10^{-2}	Low	7.33×10^{-2}	Low
	Daphnia	Daphnia magna	1000	4.568	9.85×10^{-3}	Low	8.27×10^{-3}	Low
	Algae	Scenedesmus abundans	1000	0.545	8.26×10^{-2}	Low	6.94×10^{-2}	Low

References

1. Du, Z.K.; Wang, G.W.; Gao, S.X.; Wang, Z.Y.; Aryl organophosphate flame retardants induced cardiotoxicity during zebrafish embryogenesis: by disturbing expression of the transcriptional regulators. *Aquat. Toxicol*. **2015**, *161*, 25–32. Doi: 10.1016/j.aquatox.2015.01.027.
2. Verbruggen, E.M.J.; Rila, J.P.; Traas, T.P.; Posthuma-Doodeman, C.J.A.M.; Posthumus, R.; Environmental risk limits for several phosphate esters,with possible application as flame retardant. *ChemRxiv* **2005**. http://refhub.elsevier.com/S0160-4120(13)00129-3/rf0285.
3. Cristale. J.; García Vázquez, A.; Barata, C.; Lacorte, S. Priority and emerging flame retardants in rivers: occurrence in water and sediment, Daphnia magna toxicity and risk assessment. *Environ. Int.* **2003**, *59*, 232–243. Doi: 10.1016/j.envint.2013.06.011.
4. European Commission, 2009. European Union risk assessment report: tris (2-chloroethyl) phosphate, TCEP. https://echa.europa.eu/documents/10162/2663989d-1795-44a1- 8f50-153a81133258.
5. NICNAS (National Industrial Chemicals Notification and Assessment Scheme) (2005). Priority Existing Chemical Assessment Report No. 27. URL: https://www.nicnas.gov.au/__data/assets/word_doc/0020/34832/PEC27-TBBP.docx.
